# The Penn Medicine COVID-19 Therapeutics Committee—Reflections on a Model for Rapid Evidence Review and Dynamic Practice Recommendations During a Public Health Emergency

**DOI:** 10.1093/ofid/ofad428

**Published:** 2023-08-09

**Authors:** George L Anesi, Kathleen Degnan, Lauren Dutcher, Stephen Saw, Christina Maguire, Amanda Binkley, Sonal Patel, Vasilios Athans, Todd D Barton, Shawn Binkley, Christina L Candeloro, David J Herman, Nishaminy Kasbekar, Leigh Kennedy, Jeffrey H Millstein, Nuala J Meyer, Naasha J Talati, Hinal Patel, David A Pegues, Patrick J Sayre, Pablo Tebas, Adrienne T Terico, Kathleen M Murphy, Judith A O’Donnell, Melissa White, Keith W Hamilton

**Affiliations:** Division of Pulmonary, Allergy, and Critical Care, University of Pennsylvania Perelman School of Medicine, Philadelphia, Pennsylvania, USA; Division of Infectious Diseases, University of Pennsylvania Perelman School of Medicine, Philadelphia, Pennsylvania, USA; Division of Infectious Diseases, University of Pennsylvania Perelman School of Medicine, Philadelphia, Pennsylvania, USA; Department of Pharmacy, Hospital of the University of Pennsylvania, University of Pennsylvania Health System, Philadelphia, Pennsylvania, USA; Department of Pharmacy, Penn Presbyterian Medical Center, University of Pennsylvania Health System, Philadelphia, Pennsylvania, USA; Department of Pharmacy, Penn Presbyterian Medical Center, University of Pennsylvania Health System, Philadelphia, Pennsylvania, USA; Department of Pharmacy, Hospital of the University of Pennsylvania, University of Pennsylvania Health System, Philadelphia, Pennsylvania, USA; Department of Pharmacy, Hospital of the University of Pennsylvania, University of Pennsylvania Health System, Philadelphia, Pennsylvania, USA; Division of Infectious Diseases, University of Pennsylvania Perelman School of Medicine, Philadelphia, Pennsylvania, USA; Department of Pharmacy, Hospital of the University of Pennsylvania, University of Pennsylvania Health System, Philadelphia, Pennsylvania, USA; Department of Pharmacy, Hospital of the University of Pennsylvania, University of Pennsylvania Health System, Philadelphia, Pennsylvania, USA; Division of Infectious Diseases, Penn Medicine Princeton Medical Center, University of Pennsylvania Health System, Princeton, New Jersey, USA; Department of Pharmacy, Penn Presbyterian Medical Center, University of Pennsylvania Health System, Philadelphia, Pennsylvania, USA; Division of Infectious Diseases, Pennsylvania Hospital, University of Pennsylvania Health System, Philadelphia, Pennsylvania, USA; Regional Physician Practices of Penn Medicine, Woodbury Heights, New Jersey, USA; Division of Pulmonary, Allergy, and Critical Care, University of Pennsylvania Perelman School of Medicine, Philadelphia, Pennsylvania, USA; Division of Infectious Diseases, University of Pennsylvania Perelman School of Medicine, Philadelphia, Pennsylvania, USA; Department of Pharmacy, Penn Medicine Princeton Medical Center, University of Pennsylvania Health System, Princeton, New Jersey, USA; Division of Infectious Diseases, University of Pennsylvania Perelman School of Medicine, Philadelphia, Pennsylvania, USA; Department of Medicine, University of Pennsylvania Perelman School of Medicine, Philadelphia, Pennsylvania, USA; Division of Infectious Diseases, University of Pennsylvania Perelman School of Medicine, Philadelphia, Pennsylvania, USA; Department of Pharmacy, Pennsylvania Hospital, University of Pennsylvania Health System, Philadelphia, Pennsylvania, USA; Division of Infectious Diseases, University of Pennsylvania Perelman School of Medicine, Philadelphia, Pennsylvania, USA; Division of Infectious Diseases, University of Pennsylvania Perelman School of Medicine, Philadelphia, Pennsylvania, USA; Department of Pharmacy, Penn Medicine Lancaster General Health, University of Pennsylvania Health System, Lancaster, Pennsylvania, USA; Division of Infectious Diseases, University of Pennsylvania Perelman School of Medicine, Philadelphia, Pennsylvania, USA

**Keywords:** COVID-19, emerging pathogen, evidence-based medicine, preparedness

## Abstract

The Penn Medicine COVID-19 Therapeutics Committee—an interspecialty, clinician-pharmacist, and specialist–front line primary care collaboration—has served as a forum for rapid evidence review and the production of dynamic practice recommendations during the 3-year coronavirus disease 2019 public health emergency. We describe the process by which the committee went about its work and how it navigated specific challenging scenarios. Our target audiences are clinicians, hospital leaders, public health officials, and researchers invested in preparedness for inevitable future threats. Our objectives are to discuss the logistics and challenges of forming an effective committee, undertaking a rapid evidence review process, aligning evidence-based guidelines with operational realities, and iteratively revising recommendations in response to changing pandemic data. We specifically discuss the arc of evidence for corticosteroids; the noble beginnings and dangerous misinformation end of hydroxychloroquine and ivermectin; monoclonal antibodies and emerging viral variants; and patient screening and safety processes for tocilizumab, baricitinib, and nirmatrelvir-ritonavir.

## Teaser

The Penn Medicine COVID-19 Therapeutics Committee served as a forum for rapid evidence review and the production of dynamic practice recommendations during the pandemic. We describe how the committee went about its work and how it navigated specific challenging scenarios.

As the United States anxiously awaited the arrival of the coronavirus disease 2019 (COVID-19) pandemic in early 2020, clinicians and researchers began a frantic search for guidance in how to manage patients with this novel disease—first by extrapolation from known potentially related respiratory viruses and the associated basic and clinical science literature [1], next from those on the front lines of early hot spots in China and then Italy [2–5], and then from new observational and clinical trial data emerging at a truly unprecedented pace [6]. While all of these sources hold merit, there is also a healthy skepticism to be had for early reports in a novel disease outbreak, which can be anchored on speculation and may not hold up over time [7, 8], for lessons learned from different viruses or wholly preclinical data [9–11], and for results being published through highly expedited channels, often before any peer review [12–14].

These concerns, and our institution's mandate to provide optimal care for our patients and to serve as a leader and model for COVID-19 care, motivated the formation of the Penn Medicine COVID-19 Therapeutics Committee on 11 March 2020. Over the next 3 years and continuing to the present, this committee—an interspecialty, clinician-pharmacist, and specialist–front line primary care collaboration—has served as a forum for rapid evidence review and the production of dynamic practice recommendations during the COVID-19 public health emergency, with corresponding objective improvements in outcomes for this novel disease [15]. The objectives of this article are not to report the practice recommendations that were produced, which are widely available elsewhere and still too dynamic for a static publication, but instead to describe the process by which the committee went about its work and how it navigated specific challenging and paradigm scenarios, a near-universal endeavor for hospitals in the United States and globally during the pandemic.

Our target audiences for this article are clinicians, hospital leaders, public health officials, and researchers invested in the preparedness for inevitable future threats. Our learning objectives are to discuss the logistics and challenges of forming an effective committee, undertaking a rapid evidence review process, aligning evidence-based guidelines with operational realities, and iteratively revising our recommendations in response to changing pandemic data. We specifically discuss the arc of evidence for corticosteroids; the noble beginnings and dangerous misinformation end of hydroxychloroquine and ivermectin; monoclonal antibodies and emerging viral variants; and patient screening and safety processes for tocilizumab, baricitinib, and nirmatrelvir-ritonavir.

## THE PENN MEDICINE COVID-19 THERAPEUTICS COMMITTEE

Penn Medicine—the combination of the University of Pennsylvania Health System and the University of Pennsylvania Perelman School of Medicine—includes 6 acute care hospitals that span a quaternary academic referral center, an urban teaching hospital and trauma center, and urban, suburban, and rural community-focused hospitals. These hospitals together include 6 emergency departments, >20 intensive care units, and >2900 inpatient beds accommodating annually >337 000 emergency department visits and >129 000 inpatient hospital admissions. Penn Medicine ambulatory clinics see >5.6 million outpatient visits per year. The health system serves a broad surrounding community and large referral area across urban, suburban, and rural eastern Pennsylvania and southern and central New Jersey.

The Penn Medicine COVID-19 Therapeutics Committee was formed with multidisciplinary physician and pharmacist representation from infectious diseases, pulmonary and critical care medicine, microbiology, and infection prevention and control. Committee membership included representation from all hospitals within the health system to allow for local expert input, which was helpful for aligning guidelines with operational realities and for uniform dissemination and implementation. As the pandemic progressed and therapeutics emerged from new drug classes, such as monoclonal antibodies and antivirals for early disease, the committee membership expanded to include outpatient primary care and infusion center clinicians to maintain necessary expert input across the full spectrum of COVID-19 care. The committee met weekly at the start of the pandemic, less frequently as time passed, and emergently ad hoc when immediate updates were needed, such as due to viral variant shifts and regulatory changes that added or removed agents from the US market.

Potential therapeutic agents were identified on a rolling basis selected by local and global expert opinion, extrapolation from agents with efficacy for other pathogens or with plausible mechanisms, and from the rapidly emerging COVID-19 literature (Table 1). To rapidly process, assess, and evaluate this high-volume, variable-quality data, we decentralized literature review to committee members, with one member acting as the reviewer for each candidate therapeutic. Reviewers created a summary monograph for each therapeutic agent, which included information and citations on mechanism of action, in vitro and clinical efficacy data, pharmacokinetic considerations, drug interaction concerns, dosing, monitoring parameters, and administration. Practical considerations were also included, such as procurement, supply, storage, and hospital formulary status. Reviewers created alerts through academic search engines to be notified continually of new publications and preprints. This real-time literature surveillance allowed for rapid updates to drug monographs in what amounted to a living systematic review.

**Table 1.** Selected Pharmacologic Candidates Evaluated by the Penn Medicine COVID-19 Therapeutics Committee

Abatacept
ACE inhibitors and ARBs
Anakinra
Aviptadil
Azithromycin
Baricitinib^a^
BCG vaccine
Chloroquine/hydroxychloroquine
Colchicine
Convalescent plasma^a^
Corticosteroids (dexamethasone)^a^
Dapagliflozin (SGLT2 inhibitors)
Darunavir/cobicistat
Ensovibep
Favipiravir
Fenofibrate
Fluvoxamine
Glutathione
GM-CSF
Infliximab
Interferons
Ivermectin
IVIG
Lopinavir-ritonavir
MK7110 (formerly CD24Fc)
Molnupiravir^a^
Monoclonal antibodies^b^
N-acetylcysteine
Nelfinavir
Nirmatrelvir-ritonavir^a^
Nitazoxanide
NSAIDs
Oseltamivir
Proton pump inhibitors (omeprazole)
Remdesivir^a^
Ribavirin
Ruxolitinib
Sabizabulin
Sofosbuvir-daclatasvir
Statins (HMG-CoA reductase inhibitors)
Tocilizumab/sarilumab^a^
Tofacitinib
Vitamin C
Vitamin D
Zinc

Abbreviations: ACE, angiotensin-converting enzyme; ARBs, angiotensin receptor blockers; GM-CSF, granulocyte-macrophage colony-stimulating factor; HMG-CoA, β-Hydroxy β-methylglutaryl-CoA; IVIG, intravenous immunoglobulin; NSAIDs, nonsteroidal anti-inflammatory drugs; SGLT2, sodium-glucose cotransporter 2.

a Component of current Penn Medicine treatment guidelines for coronavirus disease 2019 (at the time of publication).

b Recommendation suspended at the time of publication based on circulating severe acute respiratory syndrome coronavirus 2 viral variants.

Highlights from these monographs were curated into a master table that tracked notable updates. These master table updates, in turn, informed the discussion agenda for committee meetings at which reviewers gave evidence presentations on new therapeutic agents or existing agents with new data. Determination for guideline inclusion was by committee consensus, and specific therapeutic recommendations and revisions, including inclusion and exclusion criteria, dosing regimen, and safety monitoring, were drafted and circulated for iterative feedback. The committee was delegated authority by the standing health system Pharmacy and Therapeutics Committee to create, update, and approve health system guidelines based on these reviews. If guidelines required adding a new therapeutic agent to hospital formulary or the use of a medication with an US Food and Drug Administration (FDA) emergency use authorization (EUA), the recommendation to add that therapeutic agent was sent to the Pharmacy and Therapeutics Committee for additional approval.

To focus efforts on the highest yield therapeutic agents, the committee also had a process of retiring agents from active review when data reached a certain threshold of inefficacy, definitive trials were published, or other therapeutics were clearly preferred. However, there was a less frequent periodic ongoing review of retired agents, and in some instances, agents were unretired. For example, tocilizumab initially had data that was unfavorable, but subsequent studies demonstrated more promising safety and efficacy [16, 17]. In addition, COVID-19 convalescent plasma had poor efficacy data [18] and an unreliable supply chain; however, when the FDA EUAs for all monoclonal antibodies were revoked, additional limited convalescent plasma data emerged [19], and the supply chain improved, COVID-19 convalescent plasma was unretired for salvage use on a case-by-case basis in a very narrow group of immunosuppressed patients.

Outfacing deliverables from the committee included the web-based, publicly available “Penn Medicine COVID-19 Treatment Guidelines,” [20] a living guideline document updated in real-time throughout the pandemic. Guideline recommendations, which also served as operational workflow guides, were divided by disease severity (ie, not hospitalized, hospitalized without or with risk factors, pneumonia without hypoxemia, hypoxemia requiring low-flow supplemental oxygen, and critical illness requiring high-flow, noninvasive, and invasive respiratory support) and special populations (eg, pregnant and immunosuppressed patients).

Guideline updates were always accompanied by communication to pertinent healthcare providers, which was facilitated by the diverse committee membership. Specifically, guideline updates and revisions were announced in daily health system–wide email communications with dynamic links and further transmitted directly to highly affected service lines (eg, primary care, emergency medicine, hospital medicine, and critical care) via committee communication with service line leaders for internal dissemination. Similarly, the committee had close relationships with health system information systems and operational leadership so that medication orders and clinical decision support could be integrated rapidly into the electronic health record and instructions on ordering and administration could accompany treatment guidelines. The committee went through a standard checklist of steps when implementing novel therapeutics (Table 2).

**Table 2.** Operational Steps to Bring a New or Repurposed Drug Into Clinical Use for a Novel Pathogen

Determine whether efficacy and safety data reach threshold for clinical use
Determine how healthcare facilities will procure and share supply if relevant
Develop guidelines for local use based on available data, national guidelines, and local operational realities
Establish location of administration if relevant (eg, infusion suite, healthcare facility unit, emergency department)
Determine patient eligibility criteria (eg, proof of positive diagnostic test, underlying comorbid conditions, duration of symptoms)
Develop instructions for ordering, administration, and monitoring
Establish approval requirements for prescribers (eg, use requires infectious diseases approval, use requires antibiotic stewardship approval, prescribing is unrestricted)
Approve treatment guidelines by relevant local committee(s) or board(s) (eg, pharmacy and therapeutics committee, disease-specific therapeutics committee, medical board)
Add therapeutic agent to healthcare facility formulary and/or approve use of therapeutic agent at healthcare facility if relevant
Develop patient consent documents if relevant
Obtain local approval of consent documentation by relevant personnel (eg, office of general counsel, institutional review board)
Create standard electronic health record documentation if relevant (eg, consent documentation, patient/family discussion notes, infectious diseases and/or antibiotic stewardship approval notes)
Create electronic ordering and/or referral process in electronic health record
Create clinical decision support in electronic health record if relevant
Develop ethical allocation processes in setting of resource scarcity
Publish treatment guidelines in an accessible location for prescribers and staff (eg, website)
Educate prescribers and relevant staff on guidelines for use, approval process, prescribing instructions, and administration/monitoring instructions
Set up process to track use of therapeutic agent for public health reporting, safety monitoring, supply inventory, and guideline adherence when relevant

Serving a large health system with heterogeneous acute care hospitals and ambulatory practices and interacting frequently with city and state public health departments regarding the procurement of government-distributed therapeutics, the health system chief medical officer identified the benefit of establishing a single point of contact, which was done through the chief pharmacy officer, an infectious diseases–trained pharmacist and administrator. This single point of contact served as a longitudinal liaison between the committee, the Philadelphia Department of Public Health, the Pennsylvania Department of Health, and the Centers for Disease Control and Prevention (CDC). One critical decision made between the chief medical officer, the chief pharmacy officer, and the public health departments was the distribution of limited-supply therapeutics with FDA EUAs to a single health system hospital, which would in turn distribute internally among the other health system hospitals. This centralization allowed for internal titration of limited-supply therapeutics to meet the heterogeneous demands across our care sites. Of note, this internal sharing was possible among our Pennsylvania-based facilities but could not be extended to our New Jersey–based facilities, which were within a different state regulatory silo.

## CORTICOSTEROIDS FOR COVID-19

Among the committee's earliest recommendations was one we would soon reverse: a recommendation against routine use of corticosteroids, unselective anti-inflammatory drugs, in patients infected with severe acute respiratory syndrome coronavirus 2 (SARS-CoV-2), based on extrapolated data from other respiratory viruses. Systematic reviews and meta-analyses of influenza studies had found that corticosteroid therapy was associated with increased mortality rate, longer intensive care unit stay, and increased odds of hospital-acquired infection [21, 22]. Studies on prior outbreaks of the related coronaviruses Middle East respiratory syndrome coronavirus, and severe acute respiratory syndrome coronavirus had found that corticosteroid use was associated with delayed viral clearance [23].

Despite these concerns, there was continued interest in modulating the severe inflammation and cytokine-related lung injury that some patients with SARS-CoV-2 were developing. Many initial studies of corticosteroids in SARS-CoV-2 were small and suffered from confounding by indication, with more severely ill patients more likely to receive corticosteroids and more likely to have poorer outcomes. Our clinical practice changed dramatically in July 2020 with release of the first results from the Randomised Evaluation of COVID-19 Therapy (RECOVERY) platform trial demonstrating a significant mortality benefit of dexamethasone for patients hospitalized with SARS-CoV-2 and requiring supplemental oxygen, with the greatest benefit seen in patients requiring mechanical ventilation [24]. With this new compelling data, the committee incorporated dexamethasone therapy into our treatment guidelines with dosing, duration, and eligibility criteria based on the RECOVERY trial protocol.

Although there remain questions about optimal corticosteroid choice and dose, dexamethasone has become standard of care for hospitalized patients with severe or critical COVID-19 [25]. The corticosteroid arc within the COVID-19 saga holds multiple important lessons, including the potential fallibility of extrapolation to a novel virus even from highly related known pathogens and the humility required to swiftly reverse prior recommendations in the face of new, high-quality data that contradicts prior consensus.

## HYDROXYCHLOROQUINE AND IVERMECTIN: FROM NOBLE BEGINNINGS TO DANGEROUS MISINFORMATION

Among the earliest COVID-19 therapeutics candidates evaluated by the committee in March 2020 were hydroxychloroquine and chloroquine, immunomodulatory antiparasitic agents, based on a plausible mechanism of action and animal and in vitro data showing activity of these drugs against SARS-CoV-2 and other coronaviruses [26–36]. We reviewed an expert consensus paper from China (using online language translation) that recommended chloroquine in patients with mild and more severe COVID-19 pneumonia. There were numerous ongoing trials at the time and frequent preliminary communications about potential positive trial outcomes [37–39]. Owing to early observed COVID-19 mortality, full surge intensive care units, limited treatment options at that time, low drug cost, and easy access to the medication, our institution became early adopters of hydroxychloroquine treatment in March 2020.

During March and April 2020, we continued to review the evidence as it evolved, including our own internal observations regarding cardiac arrhythmias [40], and by the third week of April 2020, we had reviewed preliminary observational analysis and prospective randomized trials, at that stage in non–peer-reviewed databases, that suggested no efficacy benefit and some risk of harm [41–44] (citations representing subsequently published versions). On 21 April 2020, the committee concluded that it was time to remove the recommendation to use hydroxychloroquine for the treatment of COVID-19, except in the context of a clinical trial. This decision remains in effect to the present and is now supported by subsequent high-quality, large, prospective randomized trials, including those with long-term outcomes [45].

Ivermectin, another antiparasitic agent, was likewise identified as an early potential COVID-19 therapeutic candidate based on preclinical, in vitro data demonstrating reductions in SARS-CoV-2 viral RNA [46, 47], but further investigation found that plasma concentrations achieving in vitro inhibition were orders of magnitude greater than those achieved in vivo with approved dosing and studied for safety in humans [48]. With the absence of high-quality clinical studies and only a multitude of low-quality studies with clear study design issues or confounding, the committee made an initial recommendation that ivermectin be used only in the setting of a clinical trial.

As the pandemic marched on, ivermectin, alongside antivaccination efforts, became a central tenet of COVID-19 misinformation spread and believed by a swath of politicians, physicians, and members of the public. Despite its absence from every major COVID-19 treatment guideline (and indeed many overtly cautioning against its use), the dearth of high-quality supporting data, and an overt statement of no efficacy by the US manufacturer [49], ivermectin use was highly prevalent [50]. This trend and the continued cumulative evidence base motivated the strengthening of our ivermectin recommendation to state that “Ivermectin is ineffective, not recommended, and should not be prescribed or administered for the treatment or prevention of COVID-19 except as part of a well-designed clinical trial.” That recommendation remains in effect and is now further supported by every subsequent, high-quality, prospective randomized trial of ivermectin that has failed to show any clinical benefit [51–56].

## MONOCLONAL ANTIBODIES AND SARS-COV-2 VIRAL VARIANTS

Anti–SARS-CoV-2 monoclonal antibodies, which aim to bind to and neutralize viral particles, became an important, and highly dynamic, tool for the treatment of high-risk outpatients with mild-to-moderate COVID-19 infection, but their use posed significant logistical challenges [57]. Effective implementation required collaboration between the committee and leaders from the health system, pharmacy, nursing, ambulatory practices, and bioethics and legal departments. The committee first established institutional monoclonal antibody eligibility criteria for these intravenous infusions, which were in concordance with FDA EUA specifications but also included modifications to overcome regulatory ambiguities and to standardize practice across the health system, such as more specifically defining patients with immunosuppression. We then established a centralized referral process through the electronic health record for any health system providers to initiate eligibility screening. To increase access to patients in the community not previously affiliated with our health system and to improve equity in patient access, we also created a referral process for local federally qualified and city health centers through an online Research Electronic Data Capture (REDCap) form.

All monoclonal antibody referrals were screened by a centralized team to verify eligibility. When the number of referrals exceeded drug or infusion capacity, a weighted lottery was used. Created in collaboration with institutional bioethicists and following Commonwealth of Pennsylvania guidance, the lottery incorporated augmented weighting for eligible patients who were pregnant, met the Commonwealth definition of an essential worker, or resided in a location with a high area of deprivation index [58, 59]; eligible patients with a less than 6-month life expectancy had diminished weighting. In collaboration with our legal department, informed consent forms were created for each monoclonal antibody. Physicians with expertise conducted informed consent discussions via telephone with referred patients who were deemed eligible; consent was documented in the electronic health record using a standardized documentation template.

Dedicated infusion locations with trained nursing staff were created for monoclonal antibody administration so that actively infected patients would not be colocated with uninfected immunocompromised patients. A physician was available on site in infusion locations to assess any clinical emergencies. While a modest number of monoclonal antibody infusions were provided in emergency departments, a more common practice in community hospitals nationally, for most of our hospitals sustained emergency department capacity strain precluded routinely referring patients to the emergency department solely for a monoclonal antibody infusion.

Our monoclonal antibody infrastructure was modified for tixagevimab-cilgavimab [60], the dual monoclonal antibodies granted EUA for use as preexposure prophylaxis for SARS-CoV-2 infection. Given limited supply, an interdisciplinary task force, with input from clinicians, bioethicists, and legal counsel, established a prioritization scheme, giving earliest access to patients presumed to have the lowest likelihood of response to COVID-19 vaccination, based on their mechanism and timing of immunosuppression. Of note, the committee maintained, with institutional support, a consensus that tixagevimab-cilgavimab was not a substitute for vaccination and that forgoing an indicated vaccine for which one was eligible was not an indication for tixagevimab-cilgavimab prioritization. Given the intramuscular administration route and large number of patients who were, by definition, not actively infected, we decentralized tixagevimab-cilgavimab administration within specialty clinics and existing regular-use infusion locations. Finally, we also proactively identified and contacted eligible patients known to our health system, rather than relying solely on referrals, to increase access and awareness among vulnerable populations.

Unfortunately, resistance to monoclonal antibodies swiftly developed with the emergence of new SARS-CoV-2 viral variants, limiting the use of specific monoclonal antibodies at sequential points during the pandemic [61]. In response, the committee proactively assessed data on monoclonal antibody resistance and viral variant prevalence on a rolling basis, in addition to incorporating federal guidance from the FDA and the CDC. To track circulating variants, we used 2 main sources: externally, the CDC COVID Data Tracker [62], and internally, the Penn Medicine Delaware River Valley SARS-CoV-2 surveillance sequencing dashboard, which incorporated sequencing data from health system hospitals as well as the Philadelphia Department of Public Health [63, 64]. We gathered data on in vitro monoclonal antibody susceptibility from multiple sources, including FDA EUA provider fact sheets; the Stanford University Coronavirus Antiviral and Resistance Database [65], the National Institutes of Health OpenData Portal Therapeutic Activity Explorer [66], and peer-reviewed and preprint articles [61].

There were several points at which cessation of use of a particular monoclonal antibody was considered by the committee before withdrawal of the FDA EUA. In each instance, we assessed multiple factors, including regional and local prevalence of resistant variants (acknowledging data lag by several weeks) including the pattern of Philadelphia often following trends seen in New York; risks of monoclonal antibody infusion; availability of alternative monoclonal antibodies and other outpatient therapeutics, including the efficacious antiviral nirmatrelvir-ritonavir; discussion with other local institutions and public health authorities; and for tixagevimab-cilgavimab preexposure prophylaxis specifically, the possibility of future susceptible viral variants.

**Figure 1.**
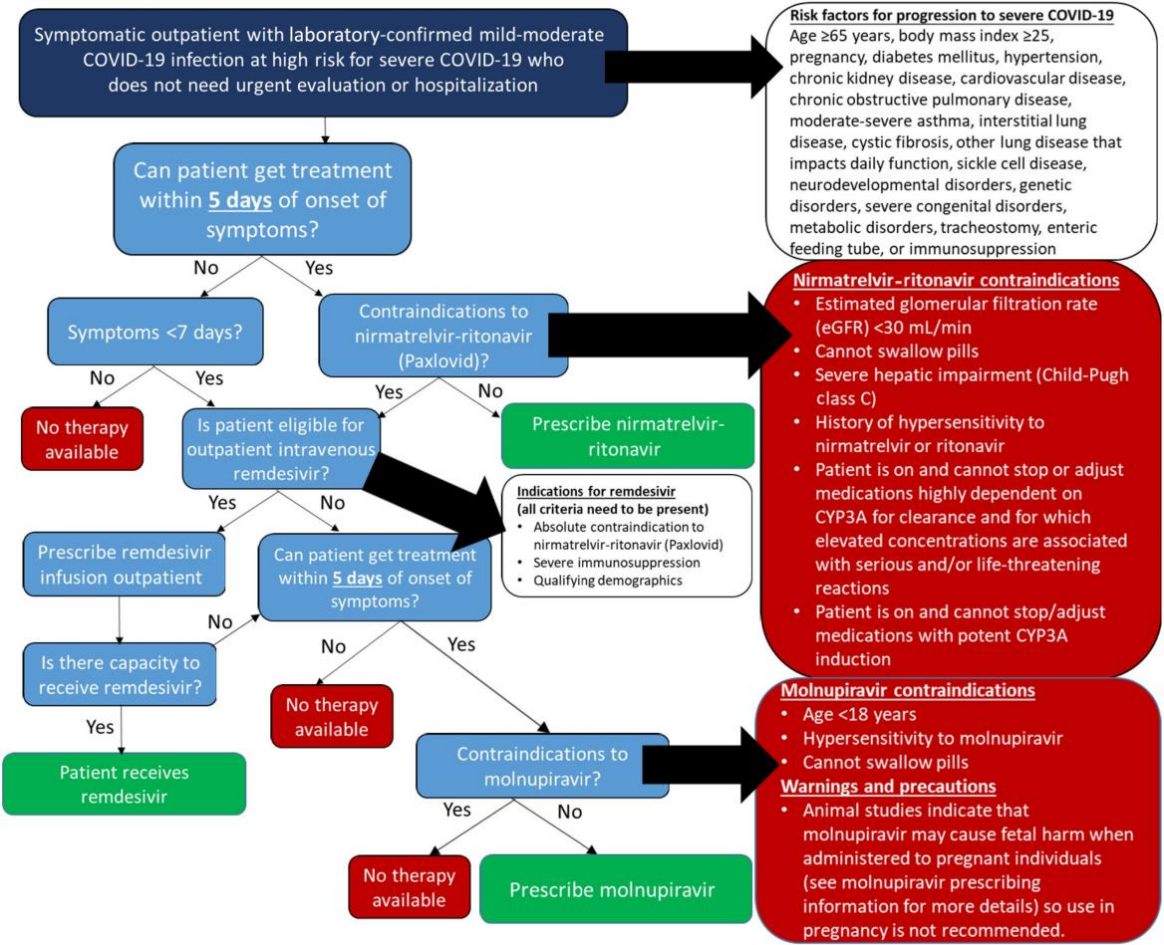
The Penn Medicine COVID-19 Therapeutics Committee outpatient treatment algorithm for coronavirus disease 2019 (COVID-18) after loss of US Food and Drug Administration (FDA) emergency use authorization for all severe acute respiratory syndrome coronavirus 2 (SARS-CoV-2) monoclonal antibodies. This algorithm was published 1 December 2022 after revocation of the FDA emergency use authorization for bebtelovimab, the last remaining available SARS-CoV-2 monoclonal antibody at that time. The algorithm assists in the consideration of ambulatory treatment with nirmatrelvir-ritonavir, molnupiravir, or remdesivir, taking into account patient-level risk factors, drug-specific contraindications, and operational realities. It is important to note that this algorithm is presented as an example of guideline communication, not as representing current recommendations, which remain dynamic. (Original publication location and current recommendations are available at https://www.uphs.upenn.edu/antibiotics/COVID19.html. CYP3A, Cytochrome P450 3A).

At the time of this writing, all once-used monoclonal antibodies for the treatment or prevention of early COVID-19—bamlanivimab with or without etesevimab, casirivimab-imdevimab, sotrovimab, bebtelovimab, and tixagevimab-cilgavimab—had withdrawal of their FDA EUA approval and ceased to be used in the United States owing to emerged resistance patterns. As additional outpatient therapeutics became available (including the antivirals nirmatrelvir-ritonavir, molnupiravir, and remdesivir), we created a recommended treatment pathway for outpatients, accounting for patient factors and availability of therapeutics, which was iteratively revised in parallel to the main treatment guidelines (Figure 1) [67].

## TOCILIZUMAB, BARICITINIB, AND NIRMATRELVIR-RITONAVIR: PATIENT SELECTION AND SAFETY

Tocilizumab, an interleukin 6 receptor antagonist, and baricitinib, a Janus kinase 1 and 2 inhibitor, have each become important adjunctive immunomodulatory agents for patients who progress to severe respiratory failure owing to SARS-CoV-2 infection. The committee outlined eligibility criteria and approaches to safety screening and monitoring informed by large clinical trials and prior literature and experience with these agents in other patient populations. The RECOVERY and COV-BARRIER trials supported the use of baricitinib, while RECOVERY and Randomized Embedded Multifactorial Adaptive Platform for Community-Acquired Pneumonia (REMAP-CAP), another large platform trial, supported the use of tocilizumab, both in combination with dexamethasone and in patients with <14 days of COVID-19 symptoms and early, rapidly progressive severe respiratory failure [68–71].

Points of debate that evolved over the pandemic included how quickly therapy must be initiated after hospitalization and the development of severe disease and how exactly to define severe disease, including intensive care unit status (admitted vs high risk of decompensation, acknowledging among-hospital differences in care processes) and degree of respiratory support (low-flow oxygen with high risk of decompensation, high-flow nasal cannula, noninvasive ventilation, or invasive mechanical ventilation). Exclusion criteria and/or dose adjustments existed for the presence and degree of renal failure, hepatic impairment, and cytopenias. The totality of evidence suggested a general preference for baricitinib, with tocilizumab available in situations where baricitinib was contraindicated or otherwise not available.

Because both agents are immunomodulatory with an infection risk, a significant case-by-case patient evaluation focused on whether or not there was a concomitant uncontrolled non–SARS-CoV-2 secondary or coinfection, such as a bacterial pneumonia. Likewise, preexisting immunosuppression was considered to be a relative, but not absolute, contraindication for both agents since these patients were excluded from clinical trials and deemed to be higher risk with additional immunomodulation. All patients were assessed for tuberculosis risk factors and hepatitis B virus serologic status. Given the pharmacologic complexity, potential adverse effect profiles, and rapidly evolving evidence base, we opted to require approval by a specialized baricitinib/tocilizumab team or an infectious diseases consultation accompanied by documentation before administration was permitted.

The ritonavir-boosted SARS-CoV-2 protease inhibitor nirmatrelvir, the first oral SARS-CoV-2 antiviral agent, was granted FDA EUA in late 2021 [72]. Despite the excitement surrounding an oral antiviral agent in the COVID-19 armamentarium, nirmatrelvir-ritonavir prescribing posed its own set of challenges including myriad drug-drug interactions (in particular due to the potent cytochrome P450 3A4 inhibition of ritonavir) and, initially, limited distribution [73]. Preexisting electronic health record drug-drug interaction alerts for chronic ritonavir were insufficient for adequately identifying high-risk alerts for immunomodulating transplant medications and also triggered unnecessary alerts with other antiretrovirals owing to short-lived duplicate therapy during the 5-day course for SARS-CoV-2.

Efforts shifted to combining information technology optimization with the use of a specialized pharmacy consultation [74]. A clinical decision support tool was designed to confirm that patients met FDA EUA criteria for use. A pharmacy consult was then recommended for any moderate-risk or high-risk drug-drug interaction. This clinical decision support was accompanied by an electronic health record revision to better select agents that required an upgrade to “high” alerts, such as transplant medications (eg, the calcineurin inhibitor tacrolimus) and the direct-acting oral anticoagulants, to ensure that the potential severity of the interaction was reflected. Pharmacy consultations used a standardized note template to document the following: no drug-drug interactions, monitoring required, adjustments needed to medications, or use of nirmatrelvir-ritonavir not recommended. When nirmatrelvir-ritonavir was safe to prescribe without any alterations, pharmacists used the federal COVID-19 Therapeutics Locator tool [75] and pended the order for the prescriber. In addition, an automatic safety stop occurred for all solid organ transplant recipients that alerted entire multidisciplinary care teams of the desire to start nirmatrelvir-ritonavir to prompt further discussions. At the time of this writing, pharmacists in our health system had performed >2000 consultations to advise on nirmatrelvir-ritonavir prescribing.

## ALIGNING GUIDELINES WITH OPERATIONAL REALITIES

A significant challenge throughout the committee's efforts was aligning optimal guidelines most supported by available evidence and the operational realities of our health system at that time. At the same time that therapeutic agents for COVID-19 provided novel treatments for an evolving pandemic, many also necessitated new processes to deliver the treatments in healthcare facilities that were already stressed by staffing, infrastructure, and resource shortages. Navigating the exponentially growing data and emerging therapeutics was only one piece. National guidelines for treatments such as monoclonal antibodies, outpatient remdesivir, and oral antivirals were often broader than could typically be fully accommodated by local capacities and drug availability. Unlike many other infectious diseases treatment guidelines, local COVID-19 therapeutic guidelines had to be informed not only by the data but also by the practicality of implementing the treatments. Eligibility criteria often needed to be adjusted based on the operational realities, usually making them more targeted to highest-risk populations.

Understanding the capacity and availability of specific therapeutic agents was essential to creating practical and sustainable treatment guidelines. This practical knowledge allowed the committee to embed eligibility criteria that prioritized patients who would benefit the most. Similarly, it facilitated setting up allocation frameworks that attempted to equitably distribute therapeutics to racially, ethnically, and socioeconomically diverse patients. In parallel, these guidelines needed to pivot rapidly as dictated by changes in data and operational realities, and these updates needed to be broadcasted to a large, complex health system, an onerous but essential process. If there was not a bidirectional flow of information between operational realities and guideline creation, guideline implementation would likely not have been as successful.

## LIMITATIONS

This article represents one health system's experience and provides a selection of discussion points but is not a comprehensive accounting of the Penn Medicine COVID-19 Therapeutics Committee's work and all related issues. In particular, we did not discuss certain therapeutic agents in depth, including the antiviral remdesivir (eg, duration of therapy, safety monitoring with impaired renal function and for bradycardia, early use in outpatients, and efficacy in critical illness), COVID-19 convalescent plasma, anakinra, and molnupiravir, among others. We also did not discuss important related issues that were governed by other groups within our health system including COVID-19 clinical education, respiratory care and critical care guidelines [15], testing policies, vaccination policies, consideration of investigational therapeutics and clinical trial enrollment, and lung transplantation.

## CONCLUSIONS

Penn Medicine would ultimately care for >20 000 patients hospitalized with COVID-19. The success of the Penn Medicine COVID-19 Therapeutics Committee in achieving a method for rapid evidence review and dynamic practice recommendations during a public health emergency was anchored in a diverse committee roster across disciplines, subspecialties and primary care, and hospitals; a commitment to, and standardized approach for, rigorous review of emerging evidence, including the explicit self-imposed mandate to revisit and revise prior recommendations based on new data; and clear attention to communication, standardization, dissemination, and version control across health system facilities. Future threats from inevitable yet-to-emerge novel pathogens may be countered with a similar or further adapted approach.

## References

[1] Paules CI , Marston HD, Fauci AS. Coronavirus infections-more than just the common cold. JAMA 2020; 323:707–8. 31971553 10.1001/jama.2020.0757

[2] Wang D , Hu B, Hu C, et al Clinical characteristics of 138 hospitalized patients with 2019 novel coronavirus-infected pneumonia in Wuhan, China. JAMA 2020; 323:1061–9. 32031570 10.1001/jama.2020.1585 PMC7042881

[3] Wu Z , McGoogan JM. Characteristics of and important lessons from the coronavirus disease 2019 (COVID-19) outbreak in China: summary of a report of 72 314 cases from the Chinese Center for Disease Control and Prevention. JAMA 2020; 323:1239–42. 32091533 10.1001/jama.2020.2648

[4] Riccardo F , Ajelli M, Andrianou XD, et al Epidemiological characteristics of COVID-19 cases and estimates of the reproductive numbers 1 month into the epidemic, Italy, 28 January to 31 March 2020. Euro Surveill 2020; 25:2000790. 10.2807/1560-7917.ES.2020.25.49.2000790 PMC7730489 33303064

[5] Spiteri G , Fielding J, Diercke M, et al First cases of coronavirus disease 2019 (COVID-19) in the WHO European region, 24 January to 21 February 2020. Euro Surveill 2020; 25:2000178. 10.2807/1560-7917.ES.2020.25.9.2000178 PMC7068164 32156327

[6] Fassin Y . Research on Covid-19: a disruptive phenomenon for bibliometrics. Scientometrics 2021; 126:5305–19. 33994601 10.1007/s11192-021-03989-w PMC8104038

[7] Abritis A , Marcus A, Oransky I. An “alarming” and “exceptionally high” rate of COVID-19 retractions? Account Res 2021; 28:58–9. 32634321 10.1080/08989621.2020.1793675

[8] Retraction Watch . Retracted coronavirus (COVID-19) papers. Available at: https://retractionwatch.com/retracted-coronavirus-covid-19-papers/. Accessed 9 March 2023.

[9] Petersen E . COVID-19 is not influenza. Lancet Respir Med 2021; 9:219–20. 33341154 10.1016/S2213-2600(20)30577-4 PMC7834708

[10] Piroth L , Cottenet J, Mariet AS, et al Comparison of the characteristics, morbidity, and mortality of COVID-19 and seasonal influenza: a nationwide, population-based retrospective cohort study. Lancet Respir Med 2021; 9:251–9. 33341155 10.1016/S2213-2600(20)30527-0 PMC7832247

[11] Rockx B , Kuiken T, Herfst S, et al Comparative pathogenesis of COVID-19, MERS, and SARS in a nonhuman primate model. Science 2020; 368:1012–5. 32303590 10.1126/science.abb7314 PMC7164679

[12] Fraser N , Brierley L, Dey G, et al The evolving role of preprints in the dissemination of COVID-19 research and their impact on the science communication landscape. PLoS Biol 2021; 19:e3000959. 10.1371/journal.pbio.3000959 PMC8046348 33798194

[13] Guterman EL , Braunstein LZ. Preprints during the COVID-19 pandemic: public health emergencies and medical literature. J Hosp Med 2020; 15:634–6. 32966198 10.12788/jhm.3491 PMC7531942

[14] Spungen H , Burton J, Schenkel S, Schriger DL. Completeness and spin of medRxiv preprint and associated published abstracts of COVID-19 randomized clinical trials. JAMA 2023; 329:1310–2. 37071105 10.1001/jama.2023.1784 PMC10114049

[15] Anesi GL , Jablonski J, Harhay MO, et al Characteristics, outcomes, and trends of patients with COVID-19-related critical illness at a learning health system in the United States. Ann Intern Med 2021; 174:613–21. 33460330 10.7326/M20-5327 PMC7901669

[16] Ghosn L , Chaimani A, Evrenoglou T, et al Interleukin-6 blocking agents for treating COVID-19: a living systematic review. Cochrane Database Syst Rev 2021; 3:CD013881. 10.1002/14651858.CD013881 PMC8406988 33734435

[17] Veiga VC , Prats J, Farias DLC, et al Effect of tocilizumab on clinical outcomes at 15 days in patients with severe or critical coronavirus disease 2019: randomised controlled trial. BMJ 2021; 372:n84. 33472855 10.1136/bmj.n84 PMC7815251

[18] De Santis GC , Oliveira LC, Garibaldi PMM, et al High-dose convalescent plasma for treatment of severe COVID-19. Emerg Infect Dis 2022; 28:548–55. 35081022 10.3201/eid2803.212299 PMC8888205

[19] Senefeld JW , Franchini M, Mengoli C, et al COVID-19 Convalescent plasma for the treatment of immunocompromised patients: a systematic review and meta-analysis. JAMA Netw Open 2023; 6:e2250647. 10.1001/jamanetworkopen.2022.50647 PMC9857047 36633846

[20] Penn Medicine . Penn Medicine COVID-19 treatment guidelines. Available at: https://www.uphs.upenn.edu/antibiotics/COVID19.html. Accessed 9 March 2023.

[21] Lansbury L , Rodrigo C, Leonardi-Bee J, Nguyen-Van-Tam J, Lim WS. Corticosteroids as adjunctive therapy in the treatment of influenza. Cochrane Database Syst Rev 2019; 2:CD010406. 10.1002/14651858.CD010406.pub3 PMC6387789 30798570

[22] Ni YN , Chen G, Sun J, Liang BM, Liang ZA. The effect of corticosteroids on mortality of patients with influenza pneumonia: a systematic review and meta-analysis. Crit Care 2019; 23:99. 30917856 10.1186/s13054-019-2395-8 PMC6437920

[23] Russell CD , Millar JE, Baillie JK. Clinical evidence does not support corticosteroid treatment for 2019-nCoV lung injury. Lancet 2020; 395:473–5. 32043983 10.1016/S0140-6736(20)30317-2 PMC7134694

[24] Collaborative Group RECOVERY . Dexamethasone in hospitalized patients with Covid-19. N Engl J Med 2021; 384:693–704. 32678530 10.1056/NEJMoa2021436 PMC7383595

[25] Kearney C , Lesieur M, Bosch NA, Walkey AJ. Corticosteroid uptake for COVID-19 after publication of RECOVERY: a difference in difference model. Ann Am Thorac Soc 2023; 20:473–6. 36525470 10.1513/AnnalsATS.202208-715RL PMC9993153

[26] Barnard DL , Day CW, Bailey K, et al Evaluation of immunomodulators, interferons and known in vitro SARS-CoV inhibitors for inhibition of SARS-CoV replication in BALB/c mice. Antivir Chem Chemother 2006; 17:275–84. 17176632 10.1177/095632020601700505

[27] Burkard C , Verheije MH, Wicht O, et al Coronavirus cell entry occurs through the endo-/lysosomal pathway in a proteolysis-dependent manner. Plos Pathog 2014; 10:e1004502. 10.1371/journal.ppat.1004502 PMC4223067 25375324

[28] Colson P , Rolain JM, Raoult D. Chloroquine for the 2019 novel coronavirus SARS-CoV-2. Int J Antimicrob Agents 2020; 55:105923. 10.1016/j.ijantimicag.2020.105923 PMC7134866 32070753

[29] de Wilde AH , Jochmans D, Posthuma CC, et al Screening of an FDA-approved compound library identifies four small-molecule inhibitors of Middle East respiratory syndrome coronavirus replication in cell culture. Antimicrob Agents Chemother 2014; 58:4875–84. 24841269 10.1128/AAC.03011-14 PMC4136071

[30] Keyaerts E , Li S, Vijgen L, et al Antiviral activity of chloroquine against human coronavirus OC43 infection in newborn mice. Antimicrob Agents Chemother 2009; 53:3416–21. 19506054 10.1128/AAC.01509-08 PMC2715625

[31] Keyaerts E , Vijgen L, Maes P, Neyts J, Van Ranst M. In vitro inhibition of severe acute respiratory syndrome coronavirus by chloroquine. Biochem Biophys Res Commun 2004; 323:264–8. 15351731 10.1016/j.bbrc.2004.08.085 PMC7092815

[32] Kono M , Tatsumi K, Imai AM, Saito K, Kuriyama T, Shirasawa H. Inhibition of human coronavirus 229E infection in human epithelial lung cells (L132) by chloroquine: involvement of p38 MAPK and ERK. Antiviral Res 2008; 77:150–2. 18055026 10.1016/j.antiviral.2007.10.011 PMC7114149

[33] Takano T , Katoh Y, Doki T, Hohdatsu T. Effect of chloroquine on feline infectious peritonitis virus infection in vitro and in vivo. Antiviral Res 2013; 99:100–7. 23648708 10.1016/j.antiviral.2013.04.016 PMC7114111

[34] Vincent MJ , Bergeron E, Benjannet S, et al Chloroquine is a potent inhibitor of SARS coronavirus infection and spread. Virol J 2005; 2:69. 16115318 10.1186/1743-422X-2-69 PMC1232869

[35] Wang M , Cao R, Zhang L, et al Remdesivir and chloroquine effectively inhibit the recently emerged novel coronavirus (2019-nCoV) in vitro. Cell Res 2020; 30:269–71. 32020029 10.1038/s41422-020-0282-0 PMC7054408

[36] Yao X , Ye F, Zhang M, et al In vitro antiviral activity and projection of optimized dosing design of hydroxychloroquine for the treatment of severe acute respiratory syndrome coronavirus 2 (SARS-CoV-2). Clin Infect Dis 2020; 71:732–9. 32150618 10.1093/cid/ciaa237 PMC7108130

[37] The National Health Commission of the People's Republic of China . Written transcript of the news briefing held by the State Council of China on Feb 17, 2020. Available at: http://www.nhc.gov.cn/xcs/yqfkdt/202002/f12a62d10c2a48c6895cedf2faea6e1f.shtml (translated from Chinese by Google Translate). Accessed 20 February 2023.

[38] Colson P , Rolain JM, Lagier JC, Brouqui P, Raoult D. Chloroquine and hydroxychloroquine as available weapons to fight COVID-19. Int J Antimicrob Agents 2020; 55:105932. 10.1016/j.ijantimicag.2020.105932 PMC7135139 32145363

[39] Gao J , Tian Z, Yang X. Breakthrough: chloroquine phosphate has shown apparent efficacy in treatment of COVID-19 associated pneumonia in clinical studies. Biosci Trends 2020; 14:72–3. 32074550 10.5582/bst.2020.01047

[40] Bhatla A , Mayer MM, Adusumalli S, et al COVID-19 and cardiac arrhythmias. Heart Rhythm 2020; 17:1439–44. 32585191 10.1016/j.hrthm.2020.06.016 PMC7307518

[41] Borba MGS , Val FFA, Sampaio VS, et al Effect of high vs low doses of chloroquine diphosphate as adjunctive therapy for patients hospitalized with severe acute respiratory syndrome coronavirus 2 (SARS-CoV-2) infection: a randomized clinical trial. JAMA Netw Open 2020; 3:e208857. 10.1001/jamanetworkopen.2020.8857 PMC12124691 32330277

[42] Magagnoli J , Narendran S, Pereira F, et al Outcomes of hydroxychloroquine usage in United States veterans hospitalized with COVID-19. Med 2020; 1:114–27.e3. 32838355 10.1016/j.medj.2020.06.001 PMC7274588

[43] Mahevas M , Tran VT, Roumier M, et al Clinical efficacy of hydroxychloroquine in patients with covid-19 pneumonia who require oxygen: observational comparative study using routine care data. BMJ 2020; 369:m1844. 32409486 10.1136/bmj.m1844 PMC7221472

[44] Tang W , Cao Z, Han M, et al Hydroxychloroquine in patients with mainly mild to moderate coronavirus disease 2019: open label, randomised controlled trial. BMJ 2020; 369:m1849. 32409561 10.1136/bmj.m1849 PMC7221473

[45] Writing Committee for the REMAP-CAP Investigators . Long-term (180-day) outcomes in critically ill patients with COVID-19 in the REMAP-CAP randomized clinical trial. JAMA 2023; 329:39–51. 36525245 10.1001/jama.2022.23257 PMC9857594

[46] Caly L , Druce JD, Catton MG, Jans DA, Wagstaff KM. The FDA-approved drug ivermectin inhibits the replication of SARS-CoV-2 in vitro. Antiviral Res 2020; 178:104787. 10.1016/j.antiviral.2020.104787 PMC7129059 32251768

[47] Heidary F , Gharebaghi R. Ivermectin: a systematic review from antiviral effects to COVID-19 complementary regimen. J Antibiot (Tokyo) 2020; 73:593–602. 32533071 10.1038/s41429-020-0336-z PMC7290143

[48] Schmith VD , Zhou JJ, Lohmer LRL. The approved dose of ivermectin alone is not the ideal dose for the treatment of COVID-19. Clin Pharmacol Ther 2020; 108:762–5. 32378737 10.1002/cpt.1889 PMC7267287

[49] Merck . Merck statement on ivermectin use during the COVID-19 pandemic. February 4, 2021. Available at: https://www.merck.com/news/merck-statement-on-ivermectin-use-during-the-covid-19-pandemic/. Accessed 11 July 2023.

[50] Chua KP , Conti RM, Becker NV. US insurer spending on ivermectin prescriptions for COVID-19. JAMA 2022; 327:584–7. 35024763 10.1001/jama.2021.24352 PMC8759024

[51] Naggie S , Boulware DR, Lindsell CJ, et al Effect of higher-dose ivermectin for 6 days vs placebo on time to sustained recovery in outpatients with COVID-19: a randomized clinical trial. JAMA 2023;329:888–97. 36807465 10.1001/jama.2023.1650 PMC9941969

[52] Bibbins-Domingo K , Malani PN. At higher dose and longer duration, ivermectin still not effective against COVID-19. JAMA 2023;329:897–8. 36805613 10.1001/jama.2023.1922

[53] Bramante CT , Huling JD, Tignanelli CJ, et al Randomized trial of metformin, ivermectin, and fluvoxamine for Covid-19. N Engl J Med 2022; 387:599–610. 36070710 10.1056/NEJMoa2201662 PMC9945922

[54] Naggie S , Boulware DR, Lindsell CJ, et al Effect of ivermectin vs placebo on time to sustained recovery in outpatients with mild to moderate COVID-19: a randomized clinical trial. JAMA 2022; 328:1595–603. 36269852 10.1001/jama.2022.18590 PMC9587497

[55] Popp M , Reis S, Schiesser S, et al Ivermectin for preventing and treating COVID-19. Cochrane Database Syst Rev 2022; 6:CD015017. 10.1002/14651858.CD015017.pub3 PMC9215332 35726131

[56] Reis G , Silva E, Silva DCM, et al Effect of early treatment with ivermectin among patients with Covid-19. N Engl J Med 2022; 386:1721–31. 35353979 10.1056/NEJMoa2115869 PMC9006771

[57] Infectious Disease Society of American (IDSA) . IDSA Guidelines on the Treatment and Management of Patients with COVID-19: Neutralizing antibodies for treatment. Available at: https://www.idsociety.org/practice-guideline/covid-19-guideline-treatment-and-management/#Neutralizingantibodiesfortreatment. Accessed 22 August 2023.

[58] Kind AJH , Buckingham WR. Making neighborhood-disadvantage metrics accessible—the neighborhood atlas. N Engl J Med 2018; 378:2456–8. 29949490 10.1056/NEJMp1802313 PMC6051533

[59] University of Wisconsin School of Medicine and Public Health . Area deprivation index v3.0. Available at: https://www.neighborhoodatlas.medicine.wisc.edu/. Accessed 9 March 2023.

[60] US Food and Drug Administration . Fact sheet for healthcare providers: emergency use authoriziation for Evusheld (tixagevimab co-packaged with cilgavimab). Available at: https://www.fda.gov/media/154701/download. Accessed 23 August 2023.

[61] Cao Y , Jian F, Wang J, et al Imprinted SARS-CoV-2 humoral immunity induces convergent Omicron RBD evolution. Nature 2023; 614:521–9. 36535326 10.1038/s41586-022-05644-7 PMC9931576

[62] Centers for Disease Control and Prevention (CDC) . COVID data tracker. Available at: https://covid.cdc.gov/covid-data-tracker/#variant-proportions. Accessed 15 March 2023.

[63] SARS-CoV-2 variants circulating in the Delaware river valley tracked by surveillance sequencing. Penn medicine. Available at: https://microb120.med.upenn.edu/data/SARS-CoV-2/). Accessed 23 February 2023.

[64] Everett J , Hokama P, Roche AM, et al SARS-CoV-2 genomic variation in space and time in hospitalized patients in Philadelphia. mBio 2021; 12:e03456-20. 10.1128/mBio.03456-20 PMC7829343 33468702

[65] Stanford University . Coronavirus Antiviral and Resistance Database. Available at: https://covdb.stanford.edu/susceptibility-data/table-mab-susc/. Accessed 22 August 2023.

[66] National Institutes of Health National Center for Advancing Translational Sciences . OpenData Portal: SARS-CoV-2 Variants & Therapeutics. Available at: https://opendata.ncats.nih.gov/variant/activity. Accessed 22 August 2023.

[67] Penn Medicine COVID-19 Therapeutics Committee. Penn Medicine COVID-19 treatment guidelines: COVID-19 outpatient medications. Available at: https://www.uphs.upenn.edu/antibiotics/COVID19/outpatient_covid19_treatment_algorithm.html. Accessed 15 March 2023.

[68] RECOVERY Collaborative Group . Baricitinib in patients admitted to hospital with COVID-19 (RECOVERY): a randomised, controlled, open-label, platform trial and updated meta-analysis. Lancet 2022; 400:359–68. 35908569 10.1016/S0140-6736(22)01109-6 PMC9333998

[69] Marconi VC , Ramanan AV, de Bono S, et al Efficacy and safety of baricitinib for the treatment of hospitalised adults with COVID-19 (COV-BARRIER): a randomised, double-blind, parallel-group, placebo-controlled phase 3 trial. Lancet Respir Med 2021; 9:1407–18. 34480861 10.1016/S2213-2600(21)00331-3 PMC8409066

[70] RECOVERY Collaborative Group . Tocilizumab in patients admitted to hospital with COVID-19 (RECOVERY): a randomised, controlled, open-label, platform trial. Lancet 2021; 397:1637–45. 33933206 10.1016/S0140-6736(21)00676-0 PMC8084355

[71] REMAP-CAP Investigators . Interleukin-6 receptor antagonists in critically ill patients with Covid-19. N Engl J Med 2021; 384:1491–502. 33631065 10.1056/NEJMoa2100433 PMC7953461

[72] Food and Drug Administration . Fact sheet for healthcare providers: emergency use authorization for Paxlovid (nirmatrelvir/ritonavir). 2023. Available at: https://www.fda.gov/media/155050/download. Accessed 20 February 2023.

[73] Anesi GL , Maguire C. Nirmatrelvir plus ritonavir for ambulatory COVID-19: expanding evidence, expanding role. Ann Intern Med 2023; 176:133–4. 36508735 10.7326/M22-3427

[74] Millstein JH , Asch DA, Hamilton K, et al Decision support and centralized pharmacy consultation for nirmatrelvir-ritonavir prescribing in an academic health system-a model to promote drug access and reduce provider burden. J Gen Intern Med 2022; 37:4028–31. 35922709 10.1007/s11606-022-07752-6 PMC9362507

[75] US Department of Health and Human Services Administration for Strategic Preparedness and Response . COVID-19 therapeutics locator. Available at: https://covid-19-therapeutics-locator-dhhs.hub.arcgis.com. Accessed 17 February 2023.

